# A prospective risk assessment of the implementation of a schistosomiasis preventive mass drug administration for children aged five years and below in the uMkhanyakude district of KwaZulu-Natal

**DOI:** 10.1186/s12913-019-4507-1

**Published:** 2019-10-07

**Authors:** Mhlengi Vella Ncube, Moses John Chimbari

**Affiliations:** School of Nursing and Public Health, College of Health Sciences, University of KwaZulu- Natal, Durban, South Africa

**Keywords:** Schistosomiasis, MDA, Risk assessment, Failure mode and effect analysis

## Abstract

**Background:**

Schistosomiasis is endemic in the uMkhanyakude district of KwaZulu-Natal, South Africa. The South Africa Department of Health (DoH) has decided to implement a schistosomiasis preventive mass drug administration program in all affected parts of the country. Quality management is part of the strategic objectives of the treatment program. We conducted a risk assessment and developed guidelines for the quality management of a schistosomiasis preventive treatment program for children aged 5 years and below in the uMkhanyakude District of KwaZulu-Natal.

**Methods:**

We conducted a scenario planning exercise by interviewing 10 child health experts from the uMkhanyakude Health District to establish potential risks associated with a planned schistosomiasis preventive control treatment program for children aged 5 years old and below. The risks were analyzed using a modified Failure Mode and Effect Analysis (FMEA). An FMEA table was produced to guide the quality management of the planned schistosomiasis preventive control treatment program for children aged 5 years and below in the uMkhanyakude Health District.

**Results:**

We identified potential risks, failure modes and possible failure corrective/preventive measures in the following activities that would be part of the mass treatment of children aged 5 years and below infected with schistosomiasis in the uMkhanyakude District. These included enrolment of children into the treatment program; general health checks; weight and height measurements; administration of drugs; reporting of side effects and monitoring and evaluation.

**Conclusion:**

We were able to use FMEA guide quality management and identify potential risks associated with the planned schistosomiasis preventive treatment program for children aged 5 years old and below in the uMkhanyakude District of KwaZulu-Natal. The FMEA for this program will be useful to the quality management of schistosomiasis preventive treatment programs for this age group in other similar settings.

## Background

Schistosomiasis is a neglected tropical disease (NTD) most prevalent in economically disadvantaged rural communities [1]. The World Health Organization (WHO) has recommended preventive mass drug administration (MDA) using praziquantel (PZQ) at a standard dose of 40 mg per kg body weight as one of the methods to control schistosomiasis in endemic areas [2]. The MDA has significantly reduced the burden of the disease in many areas such as the Hippo Valley area of Zimbabwe [3], in Sierra Leone [4], and Togo [5]. Risk mitigation to reduce medical errors is important to the success of treatment programs [6] such as MDA programs to control neglected tropical diseases (NTDs). In affluent communities, quality management prevents litigation risk due to medical errors [6], while in low resourced communities, quality management in healthcare saves valuable resources by avoiding the need for retreatment or additional resource requirements due to complications. The risk assessment, risk mitigation and quality management of a mass drug administration program requires the identification of potential problems, finding ways to detect and take corrective measures before there are major impacts on the project outcomes. The quality of treatment programs needs to be managed at clinical as well as operational levels. At a clinical level, the quality management of drug dosage, drug formulation, drug administration and management of side-effects are important [7]. At operational level, the treatment coverage risks and supply-chain efficiency need to be managed [8, 9].

Multiple factors are involved in the treatment of children with various medications, [10] including praziquantel (PZQ), the only drug approved to treat schistosomiasis, particularly in children below the age of five [11, 12]. Experimental studies have reported challenges in different stages of the treatment process [12–14]. The PZQ tablet is large and bitter [12, 15] and there is no pediatric formulation that has been approved by WHO [16]. The tablet is crushed and mixed with syrup to dim the taste to make the drug palatable to infants [12]. Despite crushing the tablet and dimming the taste, in some pre-school age children (pre-SAC) treatment studies with PZQ, children vomited the drug [11, 15]. Studies on the safety and efficacy of PZQ have reported a minimum effective dose of 20 mg/kg and maximum safe dose of 60 mg/kg in children aged below 5 years old [14, 17–19]. Under dosing with PZQ may result in partial treatment leaving the children still infected or vulnerable to schistosome infection [7]. Overdosing on the other hand causes side effects such as abdominal pains and wastage of the much needed PZQ [7]. Risk and quality management of PZQ mass administration program at a clinical level, therefore, improves overall treatment outcomes, minimises side effects, reduces the cost of managing side-effects and reduces the amount of material and financial resources required for the treatment program to be effective [20–22].

At operational level, risk of insufficient treatment coverage is common. WHO recommends a treatment coverage of at least 70% [2]. In a school based program in the KwaZulu-Natal, the coverage of the treatment program was low because some parents did not allow their children to be treated and because of unexplained absenteeism at school on the day of treatment [23]. In Burundi some people refused to participate in a treatment program after witnessing side effects on a child [24]. One of the disturbing side effects reported in children aged below five was that of a swollen body and face [11, 17]. Another operational challenge involves drug stock-outs [8]. Drug stock-out can also be a contributing factor to low treatment coverage. The problem is common in South Africa and is associated with supply chain challenges [8]. An additional challenge relating to treatment coverage and drug availability is drug delivery [9]. The specific challenges faced by an MDA program may differ from one community to another. It is important that potential mishaps that may negatively impact on the program be identified in advance and corrective measures recommended and implemented. The South Africa Department of Health (DoH) intends to implement a schistosomiasis preventive control treatment program in all affected areas in South Africa [25]. Quality management of the implementation of the schistosomiasis preventive control treatment program is a priority to the DoH [25].

The Failure Mode and Effect Analysis (FMEA) is a method that has been used in the field of engineering to predict or identify systemic problems, understand how they impact processes, project the likelihood of these problems happening and recommend corrective action [26]. FMEA has also been used to assess risk in healthcare [27]. The FMEA has the dual capabilities of identifying operational risks and facilitate quality assurance and risk mitigation; making it a good tool for developing quality management recommendations that can be designed into a system [28]. For FMEA to be performed: a multi-disciplinary team needs to be assembled; the process that needs to analyzed must be clearly defined; failure modes then have to be identified and scored based on severity and probability; indicators of the failure mode and how the failure mode will be detected have to be identified and finally corrective measures must be recommended [10, 29, 30].

We performed a proactive risk assessment of the implementation of a PZQ MDA program for children under five using a modified FMEA. Risk prediction and mitigation in a schistosomiasis preventive treatment for children below 5 years old in uMkhanyakude District, South Africa, will ensure the treatment program is efficient and of high quality.

## Methods

### Study setting and population

The study was done in uMkhanyakude Health District located in the north of KwaZulu-Natal province [31]. We selected this district because it is ranked as one of the districts that are worst affected by schistosomiasis in South Africa [32]. The district has five hospitals and 52 clinics that provide health care services to 68,000 children that are aged between 12 and 59 months [31, 33]. Each hospital services about 10 clinics and each clinic services about 1300 children [31, 33]. The health district employs experts who provide and manage different aspects of healthcare service delivery [31].

### Sampling and data collection

When conducting an FMEA, a multi-disciplinary team has to be assembled [10, 30]. In our study the multi-disciplinary team consisted healthcare professionals who are involved in a variety of healthcare programs that target different age groups including children aged 5 years old and below. We used the uMkhanyakude Health District website to identify healthcare experts that are involved in the management treatment programs targeting children aged below the age of five in the uMkhanyakude Health District. The uMkhanyakude Health District website to lists 20 professionals who are involved in the management of treatment programs. Based on our understanding of the district health system we identified 9 participants that had roles that indicated that they were involved in the management of treatment programs targeting children aged 5 years old and below. An additional participant who was not listed on the website was identified based on previous work with the participant in a similar study. Three participants were identified through snowballing. These participants were referred to us by other interviewees making the total number of identified participants to be 13. Three of the participants were not interviewed as they were not available at the times that the study was conducted. A total of 10 participants were interviewed. Out of the 10 participants 6 were engaged through face to face interviews at their place of work and 4 elected to be interviewed telephonically. All the interviews were conducted in English and by the same interviewer in order to minimize bias that may result from using two different interviewing methods. The participants had minimum qualifications of a Diploma. All participants had been working in the district for at least 2 years and therefore had sufficient knowledge of the child health activities in the district. We did not list the occupations or roles of the participants to ensure that the participants cannot be identified by inference, in adherence to the ethical requirements of the ethical regulators that approved this study. One-on-one interviews were conducted by a single interviewer at the participant’s place of work or telephonically, depending on the participant’s convenience. Written consent was provided by the experts that were interviewed orally while verbal content was provided by those that were interviewed telephonically. These methods of consent were pre-approved by the relevant research ethics committees. We asked the same questions to each participant and allowed them to provide additional details not adequately covered by the questions posed.

FMEA required the mapping of activities that are involved in the process that is being analysed [30]. Since the MDA has not been implemented, we used the scenario planning method to design interview questions. Scenario planning is a method that is used to imagine future possibilities for strategic planning [34]. The methodology identifies alternatives to address the challenges of an uncertain future [35]. We identified possible scenarios that could hinder the successful implementation of a schistosomiasis preventive treatment program for children aged 5 years and below in the uMkhanyakude District. Thus, we based the scenario planning questions on a pre-determined list of activities: a) children enrolment, b) general health assessment, c) weight and height measurements, d) dosage calculation, e) formulation of dosage, f) administration of food, g) administration of drug, and h) monitoring of side effects (Additional file 1).

We developed an interview guide for this study. Based on the interview guide that we developed we asked the participants the following: what could go wrong in the above activities; what would be the cause and impact of any the above activities not happening as planned; and what could be done to prevent or correct the mishaps that could occur during the activities (Additional file 1)? In addition to capturing interviewee responses, the interviewees responses were also recorded using a voice recorder.

### Analysis

The analysis of activities that are required in the implementation of a praziquantel mass drug administration program for children aged 5 years old and below was done using a modified Failure Mode and Effect Analysis (FMEA) [29]. Failure modes, potential causes of the failure modes, how the failure modes could be detected and recommended actions that could be taken were identified from the information that was provided during the interviews. It was not possible to include probability and severity scores because the program has not been implemented. Hence the FMEA information focused on: activities, potential failure mode, potential effects of failure, potential causes, process controls, detection point and recommended actions [29]. An FMEA table was developed using the information obtained from the interviews.

### Ethical clearance

Ethical clearance was obtained from the University of KwaZulu-Natal Biomedical Research Ethics Committee (reference number: BE403/18) and the KwaZulu-Natal Department of Health Research Committee (reference number NHRD_201809_007).

## Results

Through in-depth interviews, we identified failure modes and possible corrective actions in six activities: enrolment, general assessment, anthropometric measurements, administration of drugs, monitoring of side effects, as well as monitoring and evaluation**.**

### Enrolment failure and correction

Participants reported that some parents did not allow their children to benefit from mass treatment programs and this reduced the treatment coverage for children under 5 years old. An example of a treatment program that has experienced such a challenge is the deworming program. A participant told us that:

*“Some parents do not like the deworming treatment program …*”.

Some participants said the reason some parents do not want their children to be treated is misinformation and influence by parents whose children previously suffered side-effects due to treatment. When asked how this challenge could be addressed, the participants recommended extensive health education. They told us that traditional leaders play a very important role in health education and mobilization of the community as they command much respect. One participant said:

“If induna tells the community that my people do this, the community listens to him because they trust him.”

Other participants said at times caregivers allow their children to be treated but the children are truant. They referred to this occurrence as missed opportunities. The participants informed us that the current child health booklet, known in South Africa as the Road-to Health Booklet, makes it mandatory to record all the treatments that children aged fives old and below in the republic receive. The information in the Road-to-Health booklet makes it possible for the Ward Based Outreach Team (WBOT) or Community Care Givers (CCGs) to identify missed opportunities. If an expected dose of treatment for a particular child is missing in the booklet, that child receives the dose during an activity referred to as a “catch-up campaign”. In some cases, missed opportunities are identified during unrelated healthcare programs the child can be given the missed dose immediately or referred to clinic for treatment on a later date preferred by the caregiver.

### General health assessment and weight measurement failures

Medical history of a child is always determined during treatment programs by analyzing the child’s health booklet and asking the child and/or guardian a series of questions. A participant said it is possible for some medical history aspects like those relating to allergies not being recorded. The participant said such a mishap can be identified either when the nurse is making a decision about administering treatment or when the child experiences an adverse reaction to treatment. The participant recommended that double checking information must be encouraged during the mass treatment programs.

Some participants informed us that challenges to mass treatment programs such as a prospective praziquantel MDA could be the availability of reliable weight scales. They said while the district has a strict policy on the calibration and maintenance of medical equipment, budgetary constraints at times hinder the timely maintenance of scales or the availability of replacement scales. The participants said the nutrition program has measuring boards to measure the height of children and these boards are always available at the clinics.

### Administration of drug failure

The participants said it is common experience for children aged 5 years and below and at times even those a little older to vomit tablets for several reasons. The WBOT teams and nurses are always ready for such an eventuality. Children often vomit the tablet shortly after taking it. When asked how they would prevent this for a big and bitter tablet like PZQ, participants said they would crush the tablet and use a sweetener such as honey or syrup. The participants recommended that children should be given the crushed sweetened tablets in small quantities. In explaining this recommendation, a participant advised:

“ … when giving medicine to small children, patience is very important.”

One participant said for children who are able to swallow the tablet, they can wrap the tablet or portion of it in bread and give it to the child to swallow with liquid.

Some participants said that another challenge that may be experienced is that of drug stock outs. They said drug stock outs may be foreseen on the pharmacy’s online system by a weekly check for low stock indicators. The pharmacists may also detect drug stock outs during monthly drug stock takes. There is provision by the Department of Health for the pharmacies to apply for emergency drug stocks to avert or remedy drug stock outs.

### Side effects reporting failure

Participants in our study were of the opinion that poor reporting of side effects occur when guardians do not fully understand the treatment process. They informed us that when side effects are not reported, minor adverse effects might eventually result in health complications for the child. The participants said this could be prevented by ensuring that there is thorough health education prior to commencement of the treatment program. Guardians should be informed on how to observe side effects and be aware of how and where to report these side effects. A participant said to us:

“The clinics have a reporting form for side-effects, but you can make your own for your treatment program if you want to.”

The participants said the forms are always a useful resource for detecting and observing side effects of treatment.

### Monitoring and evaluation failures

Some participants informed us that when monitoring and evaluation involves laboratory results, challenges might occur when biological specimens are collected from an insufficient population sample size. The participants said the reasons for insufficient biological samples include: the collection not sufficiently done; logistic challenges owing to the biological specimens’ transportation vehicle breaking down; specimens being damaged as a result of inappropriate sample collection and packaging for transportation; failure to trace treated children because of poor record of caregiver contacts. The participants recommended vigilance in the maintenance of the cold chain policy and in the record keeping of caregiver contacts. A participant informed us that they have previously been able to trace children by providing names of the children to key informants in the community members.

### Scenario planning and FMEA for the MDA quality management

Using the information obtained from the interviews we used scenario planning and process analysis to identify the potential failure modes, process controls, failure detection points and recommended actions. We used scenario planning to identify possible failures process analysis to determine the order of activities in the treatment program. Table 1 shows the initial FMEA table that may be used to manage the quality of a schistosomiasis preventive mass treatment program for children aged 5 years and below in the uMkhanyakude District of KwaZulu-Natal.

**Table 1:** A suggested FMEA Document for a schistosomiasis preventive treatment program for children aged below five in the uMkhanyakude District of KwaZulu-Natal

Activity	Potential Failure Mode	Process Controls	Detection	Recommended Action(s)
Enrolment	Parents not consenting	Health education	After community dialogue.	Intensify health education. Evaluate the effect of the health education by 1. Registering children into the program shortly or soon after communicating with guardians. 2. Assessing the knowledge level of guardians relating to the program immediately after engagement.
	Child present but not treated	Roll call	Absence during treatment	Register students prior to treatment. Use register to call up children for treatment. Trace absent children and treat. Use existing structures and mechanisms to call and trace present and absent children respectively e.g. guardians of children who could not make it to crèche or ECD can be contacted by Community Care Givers to take their child to the nearest clinic.
Weight measurements	Inaccurate weight measurements	Calibration of scales	none	Use measuring boards/ dosage poles.
Administration of drugs	vomiting of medication	watching child for 30 min following treatment	By observation during the 1 h monitoring period	Administer the drug carefully and in small amounts. Wrap tablet in bread for the older children
	drug stock outs	Weekly stock check and monthly stock take.	During the weekly stock check and monthly stock take.	Communication between the monitoring and evaluation team and the District Pharmacist will result in the understanding of the demand of PZQ and the supply of adequate drug for the program. Advocating for generics and compulsory drug licensing can also help in the availability of affordable PZQ in consistently sufficient quantities.
Monitoring and evaluation	Failure to trace patient	Promptly audit the contact information before releasing the child to the guardian.	When following up patients for monitoring and evaluation.	Promptly audit the contact information before releasing the child to the guardian. Use electronic information storage such as tablets to collect the information. Tablets can be configured not to accept registrations with incomplete information. Random sampling of children for disease surveillance can be done.
	Failure to provide sample to the laboratory	Memo reminding clinics of the period when schistosomiasis cases are likely to be found and to provide laboratory samples for testing when schistosomiasis is suspected.	When compiling information for monitoring and evaluation	Localise the monitoring and evaluation activities by providing a resource for clinics to report on the laboratory outcomes for cases that were treated for schistosomiasis.
	Failure to provide sample to the laboratory	Have an emergency number for the specimen collection driver to call in the event that the vehicle is not able to reach the clinics.	When compiling information for monitoring and evaluation	Have an alternative vehicle service on standby for emergencies.
	Sample provided to the laboratory but not suitable for testing	Develop a sample evaluation report for all specimens sent to the lab by clinics. The quality of samples can be used for work appraisal.	When specimens are being processed by the laboratory.	The sample collectors need to ensure a quality check for appropriate storage on all specimens prior to collection.

## Discussion

The study aimed at identifying, detecting and preventing or correcting potential challenges that may be experienced in a PZQ MDA program for children aged 5 years old and below in the uMkhanyakude District. The method used for analysis (FMEA) has been found to be effective in the risk prediction, risk assessment,risk mitigation and quality assurance of administering pediatric drugs [10]. The FMEA is a living document that has to be reviewed consistently to record experiential information based on lessons learnt. Our data excludes specifics on the probability of experiencing the recorded challenges and also on individual side effects that can be encountered as the program has not yet been implemented. During the implementation of the MDA program, the probability of failure modes occurring and the severity of the failure modes on the treatment process or the child.

### Low treatment coverage

The risk faced by many PZQ MDA programs, irrespective of the target age group, has been low treatment coverage [36]. One of the drivers of low treatment coverage has been reported to be lack of information on the treatment and side-effects of the treatment [37]. One of the experts in the district indicated, without giving reasons, that some members of the community do not like the deworming program and may need more information on the program. In Burundi a lack of understanding on the disease and treatment caused some members of the community to refuse PZQ treatment on the understanding that if treated they would die [24]. We suggest that the challenge of lack of information could be addressed through health education. The uMkhanyakude Health District has capacity to address the health education challenges through the Health Promotions portfolio. Access to mass media like Radio Maputaland and Rise FM could be instrumental in creating a single educational dialogue throughout the district. Mass media campaigns informing communities of the schistosomiasis and the benefits of being treated may be instrumental in increasing the enrolment and treatment coverage as was observed in Kenya [38]. These campaigns and dialogues could be used to correct any misconceptions about the program [39, 40].

Accurate knowledge of schistosomiasis and the treatment program including the side-effects of treatment and how to manage them has been reported to increase the number of people participating in PZQ MDA programs [41]. In Zimbabwe, it was observed that schistosomiasis treatment coverage for school children was high when parents or guardians expressed knowledge of schistosomiasis [42]. Working with the Quality Assurance portfolio, the health promotions team could evaluate the successes and challenges of educating the community about similar programs. Collaborative work between the district’s Health Promotions Office and the Quality Assurance Office can be instrumental in mobilizing the community, through health education, to allow their children to be treated.

Another potential treatment coverage risk that the program could face is that of children with permission and present at the treatment site because of truancy to treatment. Experts in the district call this ‘Missed Opportunities’. The cause of missed opportunities is not clear and may need further investigation. Currently missed opportunities are identified by auditing the Road to Health Card to identify missed doses during well baby visits [43]. Such is the case with the deworming and vitamin A supplementation campaigns. We suggest that missed opportunities be identified using a register of the children whose parents/caregivers have consented to be compiled before the treatment begins. During treatment sessions, the children could then be identified by calling their names. Any registered children not available on the day of treatment would be looked for in the community and treated. To reduce carbon footprint of healthcare interventions, we suggest the use of electronic equipment such as tablets to develop the treatment registers. The use of electronic health information systems in resource limited setting can improve the healthcare delivery [44, 45]. The availability of open source software to electronically manage information for preventive treatment programs for diseases affecting under resourced communities will improve the efficiency of interventions in these settings [45].

### Incorrect dosing

The risk of child weight measurements being used to determine the PZQ dosing can result in the under dosing or over dosing of a child. WHO and studies on the safety and efficacy of PZQ in children aged below five recommend a PZQ dose of 40 mg/kg [2]. This dosage is supported by studies that have shown than increasing the dosage to 60 mg/kg does not significantly increase the efficacy of the drug [19, 46]. Under dosing below 40 mg/kg will reduce the efficacy of the drug and below 20 mg/kg the efficacy of the drug can be entirely lost [19]. A PZQ overdose beyond 60 mg/kg is unsafe for children aged below five [14]. It is important to ensure that proper calibration of scales before use. In resource limited setting, such as uMkhanyakude, the availability of accurate scales can be a challenge. In uMkhanyakude, replacement scales may not always be immediately available when needed due to financial constraints. The use of dosage poles has been recommended as a low maintenance alternative to the use of weight scales [13]. The uMkhanyakude health district has measuring boards that may be used for measuring height of children Measuring boards can be used to measure the length of children that are below 2 years old and the height of those that are older [47]. The measuring boards and measuring poles may be used in uMkhanyakude instead of weight scales [13]. The measuring boards and measuring poles are both accurate and expected to be easier to transport that weight scales.

Beyond the accurate calculation of doses for the children, a potential failure in treatment can happen if the child vomits the medication shortly after treatment [11, 48]. There is no pediatric formulation of PZQ on the market [49]. The PZQ tablet is bitter, large and has to be mixed with a sweetener to make it palatable for the children [16, 50]. Regardless of this effort, children do vomit the medication [11, 48]. This is expected with PZQ and reported in some studies [11, 48]. The experts in uMkhanyakude have recommended that syrup, honey or vitamin A syrup to dim the taste of PZQ. For older children, the tablet can be wrapped in bread and swallowed using with juice. Furthermore, the experts encourage that the medication be given in small amounts at a time. Given that the uMkhanyakude expert opinion is reported based on successful experience, we recommend that they follow they continue with the techniques they have mastered to administer unpalatable drugs to this age group.

### Drug stock-outs

A PZQ mass drug administration program for children aged under five could run the risk of drug stock outs. Drug stock out could happen because of challenges associated with supply chain or financial constraints. South Africa does not allow the use of PZQ generics [51] and, in the absence of donations, the country may not have enough PZQ to implement a mass administration of schistosomiasis preventive treatment [25]. We recommend the licensing of PZQ generics in order to allow for the importation of donated PZQ to be used in treatment programs. Alternatively, the possibility of compulsory drug licensing for PZQ in South Africa can be explored, based on the World Trade Organization’s agreement on Trade Related Aspects of Intellectual Property Rights (TRIPS) [52]. Drug stock-outs are common in South Africa and the National Department of Health is in the process of implementing changes to the drug supply chain system to address some of the causes of these stock outs [8]. Drug stock out could result in low treatment coverage and consequently the effect of the MDA to contribute to interrupting the transmission of schistosomiasis in this age group will be reduced. Drug stock outs could be as a result of underestimations for the demand of a drug. This can be detected by a pharmacist during a weekly stock check or monthly stock take. Drug stock outs can be remedied by making an emergency order to the depot.

### Monitoring and evaluation failure

There is a risk that treated children cannot be traced for monitoring and evaluating the MDA program. If the treated children can be traced, information that can be used to monitor the progress and understand the needs and lessons learnt for future projects will not be available when needed. It may be difficult to trace the children whose contact information is not available. In some cases, children can be traced by asking community members about the child’s home location using only the children’s names. Electronic recording of contact information could be used to address this challenge. In our experience electronic recording of information in the uMkhanyakude District has facilitated the safe storage and remote sharing of important information in a timely manner. A checking step could be added to the electronic form to ensure that all information is captured before the child is enrolled for treatment. In some cases, the child sampled for assessment could be an immigrant and impossible to trace. Parents need to be encouraged to disclose their immigration status, any potential exposure to schistosomiasis when the child’s health is being assessed [53]. This will help in the health assessment of the child and, if need be, to facilitate the remote acquiring of information for monitoring and evaluation.

The prevalence and intensity of schistosomiasis infection are important treatment effectiveness parameters that can be used to assess the impact of the MDA program to control schistosomiasis in the community [54–56]. Infection prevalence and intensity can only be determined by laboratory testing [54]. If laboratory determination of cases of schistosomiasis is not done, important treatment effectiveness assessment information will be unavailable for monitoring and evaluation. Random selection of children for disease surveillance could be routinely done to monitor and evaluate the effectiveness of the treatment program. The uMkhanyakude Health District has to provide samples for polio detection to the National Health Laboratory Services (NHLS). The samples are not always available when required. Presumptive treatment of schistosomiasis due to high likelihood of infection [57] must still have an accompanying specimen for laboratory confirmation. The same could happen to urine and stool samples for schistosomiasis detection. A memo could be provided to remind nurses to look out for schistosomiasis symptoms and to instruct them to ensure that all suspected cases of schistosomiasis are confirmed by the laboratory.

The main limitation of this study was the absence of empirical data to quantify the probability of specific failure modes occurring and to score the severity of each failure mode. This information will only be available when DoH has begun rolling out and evaluating the MDA program. Another limitation was that the information that was provided by participants was based on their experiences in programs that were not schistosomiasis preventive control programs as South Africa is yet to implement a schistosomiasis prevention MDA program. Also, none of the participants had experience working in a schistosomiasis MDA program outside South Africa. Bias may have been introduced by the interviewing the participants using two different methods, that is, some were interviewed at their place of work while others were interviewed telephonically. The bias emanating from using these two methods was reduced by having all participants being asked the same questions by a single interviewer.

## Conclusion

Implementing a schistosomiasis preventive treatment program for children aged years old and below in the uMkhanyakude District should identify and mitigate for operational risks that may compromise the quality of the program. We have identified some of the major risks and recommended action that should be taken. The risks identified are: low treatment coverage, incorrect dosing, vomiting of drug, drug stock outs and unavailability of data for monitoring and evaluation. The recommendations provided in the suggested FMEA document (Table 1) may be adopted for use as the initial quality management tool. The FMEA document can expand in scope and depth as experience develops in the implementation of the envisaged schistosomiasis preventive control MDA for children aged 5 years and below in the uMkhanyakude District. We recommend that the final FMEA document be a living document with empirical information added post the initial stages of the program. The findings of this study will be shared with the uMkhanyakude Health District authorities and the National Department of Health in South Africa. The findings and recommendation from our study may apply to similar poor resource settings beyond South Africa.

## Supplementary information

Additional file 1: Interview guide. FMEA Interview Guide for Pediatric PZQ Administration in uMkhanyakude Health District. The interview guide that was used to obtain information for the FMEA of a prospective Schistosomiasis Preventive Mass Drug in the uMkhanyakude District of KwaZulu-Natal. (DOCX 14 kb)

## Data Availability

Data will be made available upon request from the corresponding author.

## Abbreviations

CCG: Community Care Giver
DoH: The South Africa Department of Health
ECD: Early Childhood Development
FMEA: Failure Mode and Effect Analysis
MDA: Mass Drug Administration
NHLS: National Health Laboratory Services
NTD: Neglected Tropical Diseases
PZQ: Praziquantel
SCI: Schistosomiasis Control Initiative
STH: Soil Transmitted Helminths
TB: Tuberculosis
TRIPS: Trade Related Aspects of Intellectual Property Rights
UKZN: University of KwaZulu-Natal
WBOT: Ward Based Outreach Teams
WHO: World Health Organisation

## References

[1] King CH (2010). Parasites and poverty: the case of schistosomiasis. Acta Trop.

[2] WHO (2018). WHO. Schistosomiaisis Control and preventive chemotherapy.

[3] Chimbari M, Ndlela B (2001). Successful control of schistosomiasis in large sugar irrigation estates of Zimbabwe. Cent Afr J Med.

[4] Wang W, Liang Y (2014). Mass drug administration (MDA) for schistosomiasis. J Infect Dis.

[5] Bronzan RN (2018). Impact of community-based integrated mass drug administration on schistosomiasis and soil-transmitted helminth prevalence in Togo. PLoS Negl Trop Dis.

[6] Chiozza ML, Ponzetti C (2009). FMEA: a model for reducing medical errors. Clin Chim Acta.

[7] Palha De Sousa CA (2014). Dosing of Praziquantel by Height in Sub-Saharan African Adults. Am J Trop Med Hyg.

[8] Bateman C (2013). Drug stock-outs: inept supply-chain management and corruption. S Afr Med J.

[9] Tuhebwe D (2015). Uptake of mass drug administration Programme for schistosomiasis control in Koome Islands, Central Uganda. PLOS ONE.

[10] Lago P (2012). Use of FMEA analysis to reduce risk of errors in prescribing and administering drugs in paediatric wards: a quality improvement report. BMJ Open.

[11] Mutapi F (2011). Schistosoma haematobium treatment in 1–5 year old children: safety and efficacy of the antihelminthic drug praziquantel. PLoS Negl Trop Dis.

[12] Navaratnam AMD (2012). Efficacy of praziquantel syrup versus crushed praziquantel tablets in the treatment of intestinal schistosomiasis in Ugandan preschool children, with observation on compliance and safety. Trans R Soc Trop Med Hyg.

[13] Sousa-Figueiredo JC, Betson M, Stothard JR (2012). Treatment of schistosomiasis in African infants and preschool-aged children: downward extension and biometric optimization of the current praziquantel dose pole. Int Health.

[14] Olliaro PL (2013). Practical dosing of praziquantel for schistosomiasis in preschool-aged children. Tropical Med Int Health.

[15] Sousa-Figueiredo J (2012). Performance and safety of Praziquantel for treatment of intestinal schistosomiasis in infants and preschool children. PLoS Negl Trop Dis.

[16] Mduluza T, Mutapi F (2017). Putting the treatment of paediatric schistosomiasis into context. Infect Dis Poverty.

[17] Coulibaly JT (2012). Efficacy and safety of praziquantel in preschool-aged children in an area co-endemic for *Schistosoma mansoni* and *S. haematobium*. PLoS Negl Trop Dis.

[18] Coulibaly JT (2017). Efficacy and safety of praziquantel in preschool-aged and school-aged children infected with Schistosoma mansoni: a randomised controlled, parallel-group, dose-ranging, phase 2 trial. Lancet Glob Health.

[19] Coulibaly JT (2018). Efficacy and safety of ascending doses of praziquantel against Schistosoma haematobium infection in preschool-aged and school-aged children: a single-blind randomised controlled trial. BMC Med.

[20] Jeve YB (2018). Raising quality whilst reducing cost in health care: a retrospective cohort study. Int J Health Plann Manag.

[21] Andel C (2012). The economics of health care quality and medical errors. J Health Care Finance.

[22] Lassetter JH, Warnick ML (2003). Medical errors, drug-related problems, and medication errors: a literature review on quality of care and cost issues. J Nurs Care Qual.

[23] Randjelovic A (2015). A study of hurdles in mass treatment of schistosomiasis in KwaZulu-Natal, South Africa. S Afr Fam Pract.

[24] Ndayishimiye O (2014). Control of neglected tropical diseases in Burundi: partnerships, achievements, challenges, and lessons learned after four years of Programme implementation. PLoS Negl Trop Dis.

[25] Health Do. South Africa National Master Plan for the Elimination of Neglected Tropical Diseases *(2019–2025)*. Johannesburg: South African Department of Health; 2019.

[26] Schriefer J, Leonard MS (2012). Patient safety and quality improvement: an overview of QI. Pediatr Rev.

[27] Liu Hu-Chen (2019). FMEA for Proactive Healthcare Risk Analysis: A Systematic Literature Review. Improved FMEA Methods for Proactive Healthcare Risk Analysis.

[28] Shaughnessy EE (2017). Quality improvement feature series article 1: introduction to quality improvement. J Pediatric Infect Dis Soc.

[29] Ashley L (2010). A practical guide to failure mode and effects analysis in health care: making the Most of the team and its meetings. Jt Comm J Qual Patient Saf.

[30] Ookalkar AD (2009). Quality improvement in haemodialysis process using FMEA. Int J Qual &; Rel Manag.

[31] KZNHealth (2018). UMkhanyakude Health District.

[32] Kabuyaya M (2017). Efficacy of praziquantel on Schistosoma haematobium and re-infection rates among school-going children in the Ndumo area of uMkhanyakude district, KwaZulu-Natal, South Africa. Infect Dis Poverty.

[33] Health Do (2017). Province of KwaZulu-Natal Annual Report 2016/2017 Vote 7.

[34] Amer M, Daim TU, Jetter A (2013). A review of scenario planning. Futures.

[35] Coates JF (2000). Scenario planning. Technol Forecast Soc Chang.

[36] WHO (2013). Schistosomiasis Progress Report (2001–2011) and Strategic Plan (2012–2020).

[37] Burnim M, Ivy JA, King CH (2017). Systematic review of community-based, school-based, and combined delivery modes for reaching school-aged children in mass drug administration programs for schistosomiasis. PLoS Negl Trop Dis.

[38] Omedo M (2014). The effect of a health communication campaign on compliance with mass drug administration for schistosomiasis control in western Kenya—the SCORE project. Am J Trop Med Hyg.

[39] Chami GF (2017). Community-directed mass drug administration is undermined by status seeking in friendship networks and inadequate trust in health advice networks. Soc Sci Med.

[40] Gholami M (2017). Evaluation of the impact of a mass media campaign on periodontal knowledge among Iranian adults: a three-month follow-up. PLoS One.

[41] Muhumuza S (2013). Uptake of preventive treatment for intestinal schistosomiasis among school children in Jinja district, Uganda: a cross sectional study. PLoS One.

[42] Chimbari Moses J. (2012). Enhancing Schistosomiasis Control Strategy for Zimbabwe: Building on Past Experiences. Journal of Parasitology Research.

[43] Sokhela D, Sibiya M, Gwele N (2018). Monitoring well-baby visits in primary healthcare facilities in a middle-income country. S Afr J Child Health.

[44] Were MC (2010). Evaluating a scalable model for implementing electronic health records in resource-limited settings. J Am Med Inform Assoc.

[45] Millard PS, Bru J, Berger CA (2012). Open-source point-of-care electronic medical records for use in resource-limited settings: systematic review and questionnaire surveys. BMJ Open.

[46] Kabuyaya M, Chimbari MJ, Mukaratirwa S (2018). Efficacy of praziquantel treatment regimens in pre-school and school aged children infected with schistosomiasis in sub-Saharan Africa: a systematic review. Infectious diseases of poverty.

[47] Corsi DJ, Mejía-Guevara I, Subramanian SV (2016). Risk factors for chronic undernutrition among children in India: estimating relative importance, population attributable risk and fractions. Soc Sci Med.

[48] Nalugwa A (2015). Single versus double dose Praziquantel comparison on efficacy and Schistosoma mansoni re-infection in preschool-age children in Uganda: a randomized controlled trial. PLoS Negl Trop Dis.

[49] Montresor A, Garba A (2017). Treatment of preschool children for schistosomiasis. Lancet Glob Health.

[50] Osakunor DNM, Woolhouse MEJ, Mutapi F (2018). Paediatric schistosomiasis: what we know and what we need to know. PLoS Negl Trop Dis.

[51] Magaisa K (2015). A review of the control of schistosomiasis in South Africa. S Afr J Sci.

[52] WTO. 2006; Available from: https://www.wto.org/english/tratop_e/trips_e/pharma_ato186_e.htm. Accessed 18 Jan 2019.

[53] Blach O (2011). An outbreak of schistosomiasis in travellers returning from endemic areas: the importance of rigorous tracing in peer groups exposed to risk of infection. J Public Health.

[54] Toor J (2018). The design of schistosomiasis monitoring and evaluation programmes: the importance of collecting adult data to inform treatment strategies for Schistosoma mansoni. PLoS Negl Trop Dis.

[55] Turner HC (2017). Economic considerations for moving beyond the Kato-Katz technique for diagnosing intestinal parasites as we move towards elimination. Trends Parasitol.

[56] Michael E, Meyrowitsch D, Simonsen P (1996). Cost and cost effectiveness of mass diethylcarbamazine chemotherapy for the control of bancroftian filariasis: comparison of four strategies in Tanzania. Tropical Med Int Health.

[57] Flagg EW (2007). High prevalence and presumptive treatment of schistosomiasis and Strongyloidiasis among African refugees. Clin Infect Dis.

