# Supplementary material for: A prospective risk assessment of the implementation of a schistosomiasis preventive mass drug administration for children aged five years and below in the uMkhanyakude district of KwaZulu-Natal
Source: BMC Health Serv Res. 2019 Oct 7;19:685. doi: 10.1186/s12913-019-4507-1 (PMC6781343; doi:10.1186/s12913-019-4507-1)
Supplement: Supplementary file 1 — Interview guide. FMEA Interview Guide for Pediatric PZQ Administration in uMkhanyakude Health District. The interview guide that was used to obtain information for the FMEA of a prospective Schistosomiasis Preventive Mass Drug in the uMkhanyakude District of KwaZulu-Natal. (DOCX 14 kb) [file 12913_2019_4507_MOESM1_ESM.docx]

**FMEA Interview Guide for Pediatric PZQ Administration in uMkhanyakude Health District.**

What activities do you consider important for treatment of schistosomiasis in children aged below the age of five? Participant provide answers that may be categorized as follows.

| 1. Receiving child |
| --- |
| 1. General health assessment |
| 1. Weight/Height measurement |
| 1. Dosage calculation |
| 1. Formulation of dosage 2. Administration of food |
| 1. Administration of drug |

The following questions based on identified categories are posed as discussion points (table 1):

1. What could go wrong in each activity?
2. What would be the cause and impact of the failures in the activities?
3. How often would you anticipate these failures to occur?
4. What measures do you recommend to be taken; when and by whom to prevent or correct these failures?
5. Is there anything relating to quality management, quality improvement or quality control that you would like to tell us about.

**Table 1: FMEA interview questions**

| **Interview Questions** | |
| --- | --- |
| **Main Question** | **Follow up question** |
| 1. What could go wrong during? (*insert activity*).  *Write notes on that which could go wrong during the activity*) | Please describe how (insert activity) could go wrong? |
| 2. What would cause (*insert potential failure modes mentioned in question one individually*)? |  |
| 3. Who is most likely to detect (insert potential failure modes mentioned in question one individually) ? | At what point in the treatment process is/are *(insert role of the individuals mentioned in response to question 3)* likely to detect that *(insert potential failure modes mentioned in question one individually*) has happened? |
| 4. What could be the impact of cause (*insert potential failure modes mentioned in question one individually*)? |  |
| 5. Can (*insert potential failure modes mentioned in question one individually*) be prevented or corrected? |  |
| 1. How can (*insert potential failure modes that were classified as preventable, individually)* be prevented? | At what stage in the treatment process could *(insert potential failure modes that were classified as preventable, individually)* be prevented?  Who is most likely to be in a position to prevent (insert potential failure modes that were classified as preventable, individually). |
| 1. How can (insert potential failure modes that were classified as correctable, individually) be correctable? | Does the correction reverse or does it alleviate (*insert potential failure modes that were classified as correctable, individually)* |
| 1. What is the likelihood of (*insert potential failure modes mentioned in question one individually*) to occur? |  |
| 1. What other quality control, quality management of quality improvement recommendations relating to child treatment programs are you willing to share with us? |  |
